# Toward Integrative Biomechanical Models of Osteochondral Tissues: A Multilayered Perspective

**DOI:** 10.3390/bioengineering12060649

**Published:** 2025-06-13

**Authors:** Bruna Silva, Marco Domingos, Sandra Amado, Juliana R. Dias, Paula Pascoal-Faria, Ana C. Maurício, Nuno Alves

**Affiliations:** 1 Centre for Rapid and Sustainable Product Development (CDRSP), Polytechnic of Leiria, 2430-028 Marinha Grande, Portugal; sandra.amado@ipleiria.pt (S.A.); juliana.dias@ipleiria.pt (J.R.D.); paula.faria@ipleiria.pt (P.P.-F.); 2 Associate Laboratory for Advanced Production and Intelligent Systems (ARISE), 4200-465 Porto, Portugal; 3 Department of Mechanical and Aerospace Engineering, School of Engineering, Faculty of Science and Engineering & Henry Royce Institute, The University of Manchester, Manchester M13 9PL, UK; marco.domingos@manchester.ac.uk; 4 Department of Mathematics, School of Technology and Management, Polytechnic of Leiria, 2411-901 Leiria, Portugal; 5 Centro de Estudos de Ciência Animal (CECA), Instituto de Ciências, Tecnologias e Agroambiente da Universidade do Porto (ICETA), 4050-453 Porto, Portugal; ana.colette@hotmail.com; 6 Departamento de Clínicas Veterinárias, Instituto de Ciências Biomédicas de Abel Salazar (ICBAS), Universidade do Porto (UP), 4099-002 Porto, Portugal; 7 Associate Laboratory for Animal and Veterinary Science (AL4AnimalS), 1300-477 Lisboa, Portugal; 8 Department of Mechanical Engineering, School of Technology and Management, Polytechnic of Leiria, 2411-901 Leiria, Portugal

**Keywords:** joint tissues, osteochondral modeling, articular cartilage, tidemark, calcified cartilage, subchondral bone, trabecular bone, constitutive models

## Abstract

Understanding the complex mechanical behavior of osteochondral tissues in silico is essential for improving experimental models and advancing research in joint health and degeneration. This review provides a comprehensive analysis of the constitutive models currently used to represent the different layers of the osteochondral region, from articular cartilage to subchondral bone, including intermediate regions such as the tidemark and the calcified cartilage layer. Each layer exhibits unique structural and mechanical properties, necessitating a layer-specific modeling approach. Through critical comparison of existing mathematical models, the viscoelastic model is suggested as a pragmatic starting point for modeling articular cartilage zones, the tidemark, and the calcified cartilage layer, as it captures essential time-dependent behaviors such as creep and stress relaxation while ensuring computational efficiency for initial coupling studies. On the other hand, a linear elastic model was identified as an optimal starting point for both the subchondral bone plate and the subchondral trabecular bone, reflecting their dense and stiff nature, and providing a coherent framework for early-stage multilayer integration. This layered modeling approach enables the development of physiologically coherent and computationally efficient representations of osteochondral region modeling. Furthermore, by establishing a layer-specific modeling approach, this review paves the way for modular in silico simulations through the coupling of computational models. Such an integrative framework supports scaffold design, in vitro experimentation, preclinical validation, and the mechanobiological exploration of osteochondral degeneration and repair. These efforts are essential for deepening our understanding of tissue responses under both physiological and pathological conditions. Ultimately, this work provides a robust theoretical foundation for future in silico and in vitro studies aimed at advancing osteochondral tissue regeneration strategies.

## 1. Introduction

Osteoarthritis (OA) is the most common form of arthritis, affecting approximately 7.6% of the global population (about 595 million people) as of 2020 [1]. While projections estimate a marked increase in OA prevalence by 2050, with knee OA expected to rise by 74.9%, hip OA by 78.6%, and other forms by 95.1%, this trajectory is not inevitable. Modifiable risk factors, such as obesity, sedentary behavior, and occupational stress, significantly contribute to disease onset and progression. Preventative interventions targeting these factors may mitigate the projected burden of OA [1].

To effectively tackle this challenge, it is crucial to deepen our understanding of joint biomechanics, particularly through the comprehensive characterization of osteochondral (OC) tissues, which are directly implicated in joint health and disease progression [2,3].

The biomechanical characterization of OC tissues has increasingly become a focal area of scientific research, given their essential role in preserving the functional integrity of synovial joints, especially in pathological contexts such as OA and traumatic OC lesions [2,4]. The OC unit comprises articular cartilage (AC) and subchondral bone (SB), each exhibiting distinct biomechanical properties that together facilitate appropriate mechanical load distribution and protect joint integrity [5].

Although numerous studies have investigated AC and SB independently, a recent systematic review by Berni et al. highlighted a critical gap in the literature: the lack of studies examining the OC unit as a single functional entity [6]. This is a limitation, given that cartilage and SB possess distinct biomechanical properties that, together, ensure the effective distribution of load and preserve joint integrity [5]. Importantly, the biomechanical interplay between these tissues cannot be fully understood through isolated analyses alone. For instance, alterations in SB stiffness have been shown to directly influence the mechanical and structural properties of the overlying cartilage, thereby contributing to the progression of joint degeneration [2,7].

Given this complexity, there is a compelling need for robust experimental protocols and sophisticated mathematical models capable of realistically simulating the integrated biomechanical response of OC tissues. Advanced mathematical models, such as biphasic and triphasic poroelastic models for cartilage [8,9], and viscoelastic and elastoplastic models for bone [10,11], have already proven effective in accurately describing specific characteristics of these tissues. Nonetheless, achieving the coherent and experimentally validated integration of these models into a unified representation remains a significant methodological challenge [12].

The effective integration of these biomechanical models can improve clinical practice by facilitating early and precise diagnosis, allowing tailored therapeutic strategies, and enhancing patient-specific outcomes. Ultimately, this approach could contribute to reduced healthcare costs and better quality of life for individuals affected by OA [1,13].

This paper aims to (a) analyze the layered architecture of osteochondral (OC) tissue, with particular focus on the intermediate regions between cartilage and bone, in order to understand their structural behavior and role in load transfer; (b) explore the constitutive models currently used to simulate the mechanical response of each layer, evaluating their capacity to represent the complexity of OC tissues; and (c) build upon this analysis to propose a layer-specific modeling perspective that reflects the unique characteristics of each region.

This modular strategy provides a physiologically coherent and computationally manageable foundation for future studies, with the final goal of coupling these individual models into an integrated framework capable of simulating the full biomechanical behavior of the OC unit under both physiological and pathological conditions.

## 2. Structural and Functional Organization of the Osteochondral Unit

**Figure 1:**
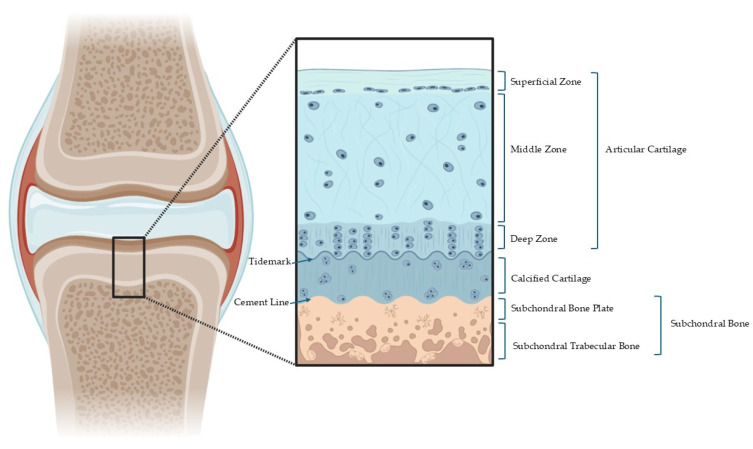
Schematic representation of OC tissue layered structure.

The OC structure exhibits a multilayered organization, as schematically illustrated in Figure 1. This complex architecture includes the AC, tidemark, calcified cartilage layer (CCL), and SB, each contributing uniquely to the biomechanical function and overall performance of the joint through their specialized roles and interactions [5]. This review primarily addresses the osteochondral structure of load-bearing synovial joints such as the knee and hip. Small joints, mandibular condyles, and vertebral facets possess distinct anatomical and mechanical characteristics that fall outside the scope of this analysis. The quantitative mechanical properties of OC tissues are summarized in Table 1.

### 2.1. Articular Cartilage

The AC is composed of three structurally and functionally distinct zones [14,15,16]. The superficial zone contains collagen fibers aligned parallel to the articular surface, providing resistance to shear forces. The middle zone features a more irregular collagen network with abundant proteoglycans, enabling the absorption and distribution of compressive loads. In the deep zone, collagen fibers are oriented perpendicular to the surface, anchoring the cartilage to the calcified layer and supporting high compressive loads during dynamic activities such as running or jumping [14,15,16].

The AC exhibits viscoelastic behavior, essential for absorbing and distributing applied forces, allowing the cartilage to deform under load and return to its original shape once the load is removed. This behavior is crucial for minimizing stress on the joints and for shock absorption. However, with aging, the ability of chondrocytes to maintain and repair the matrix decreases, leading to an increased susceptibility to degeneration [14,15,16].

### 2.2. Tidemark

The tidemark is a clear histological demarcation indicating a transition from non-calcified to calcified cartilage. It was initially identified by Fawns and Landells in 1953 [17] and later described in detail in the patellar bone by Gannon and Sokoloff [18], as an undulating hematoxyphilic line, up to 10 μm thick, visible under light microscopy. In spite of early reports, the tidemark is no longer regarded as a passive morphological boundary but rather as a three-dimensional, metabolically active structure that penetrates into the CCL and interdigitates with underlying bone and marrow spaces [12,19].

Structurally, the tidemark consists of a band of fibrils attached to collagen fibers anchored in the mineral-poor cartilage layer, which twist into organized bundles that extend through the CCL and ultimately integrate into the SB [15,20]. This anatomical configuration provides the cartilage with increased resistance to shear forces, preventing its separation from the bone [19].

Mechanically, the tidemark delineates the mineralization front and marks a significant transition in tissue stiffness. It has been shown to anchor unmineralized collagen fibrils and regulate force transmission between the compliant AC and the stiffer CCL [21,22]. Under static compressive loads, AC deformation often terminates at the tidemark, suggesting its role as a mechanical buffer that modulates stress distribution. Furthermore, collagen fiber orientation and packing density within the tidemark region adapt to loading conditions, reinforcing its function in stress absorption and transmission [22].

### 2.3. Calcified Cartilage Layer

The CCL, typically ranging from 20 to 250 µm thick, is a distinct region situated between the tidemark (upper boundary) and cement line (lower boundary) [12,23]. It is composed of hypertrophic chondrocytes embedded in a mineralized extracellular matrix containing type II collagen (approximately 20%) and hydroxyapatite (approximately 65%) by dry weight, biochemically positioning it between hyaline cartilage and the SB [12,23].

Structurally, the CCL anchors collagen fibrils originating in the deep zone of the AC into the subchondral bone, forming a robust transitional interface [12]. Collagen fibers within the CCL are oriented perpendicular to the articular surface and become structurally integrated with osteoid produced by osteoblasts at the bone interface, forming a mechanically robust transition [24].

These hypertrophic chondrocytes represent a terminal differentiation stage rather than transient osmotic swelling. They are typically associated with increased alkaline phosphatase activity and matrix mineralization, marking the final phase of the chondrogenic lineage [24]. This phenotype is consistent with observations in the CCL, where chondrocytes contribute to matrix calcification and express markers of hypertrophy, such as type X collagen and alkaline phosphatase [12,24].

Despite its thinness, this layer exhibits unique mechanical properties: it is stiffer than the overlying cartilage—estimated to be 10 to 100 times stiffer than hyaline cartilage—but remains about one-tenth as stiff as bone [25]. This intermediate stiffness plays a crucial role in modulating stress transfer across the mechanically heterogeneous osteochondral interface, dissipating loads, preventing stress concentrations, and reducing the propagation of microcracks at the cartilage–bone junction [12].

Its complex microarchitecture, marked by low cell density, hypertrophic chondrocytes, and irregular collagen orientations, further enhances shear resistance and mechanical stability at the OC junction [12,15]. Additionally, the CCL contains nanochannels and micropores that allow limited diffusion of solutes, contributing to controlled biochemical exchange while preventing vascular invasion into the hyaline cartilage [26,27].

From a modeling perspective, the distinct mechanical and structural features of the CCL significantly influence the biomechanical behavior of the OC unit. The CCL plays a key role in dissipating loads and smoothing stress transfer at the cartilage–bone interface, justifying its inclusion as an independent layer in mechanical simulations [12]. Even modest changes in its stiffness or anisotropy can alter local strain and pressure distributions, as shown in cartilage models and applicable to this transitional region [28]. In addition, microstructural characteristics such as collagen orientation and porosity affect solute transport and interfacial mechanics, further supporting the need for realistic CCL representation in computational frameworks [26]. Experimental studies with engineered scaffolds also confirm that mimicking CCL properties improves stress distribution and reduces failure risk at the cartilage–bone junction [23]. Together, these findings demonstrate that accurately modeling the CCL is essential to capture physiologically relevant load transfer and enhance the predictive value of osteochondral simulations.

### 2.4. Subchondral Bone

The SB plays a critical role in absorbing and distributing mechanical loads, maintaining joint architecture, and anchoring the OC complex to the underlying skeletal structure. Structurally, the SB is composed primarily of type I collagen and a highly mineralized matrix, with hydroxyapatite accounting for approximately 85% of its dry weight [29]. This composition imparts a stiffness of approximately 4 GPa, significantly greater than that of the overlying cartilage layers. Unlike the adjacent CCL, the SB does not express type II collagen, highlighting a distinct phenotypic and functional shift at the OC interface. Its matrix is relatively porous and contains little collagen, features that facilitate vascularization and interaction with the bone marrow.

Anatomically, the SB is composed of two distinct regions with specific structural and functional characteristics: a subchondral bone plate (SBP) and a deeper layer of subchondral trabecular bone (STB). These compartments differ in mechanical properties, mineral composition, porosity, and biological activity [3,30].

#### 2.4.1. Subchondral Bone Plate

The SBP is a thin and dense layer that typically measures around 0.8 mm in thickness, though this may vary depending on anatomical location, age, and mechanical loading [3]. Structurally, the SBP is characterized by low porosity, high mineral density, and limited metabolic activity [3,31]. It forms a critical component of the OC junction, providing a stable interface between the AC and the underlying trabecular bone [3]. Functionally, it serves as a mechanically efficient support layer, facilitating the transmission and dissipation of compressive loads to the STB [3,30]. In this physiological state, the SBP remains avascular and nerveless, acting as a barrier between the synovial environment and the subchondral bone, thereby contributing to the maintenance of joint homeostasis [3].

#### 2.4.2. Subchondral Trabecular Bone

The STB lies beneath the cortical plate and is composed of a porous, lattice-like network of interconnected trabeculae. This region plays a critical biomechanical role by distributing loads transmitted from the SBP over a wider area, damping impact forces, and contributing to the shock-absorbing capacity of the OC unit. In addition to its mechanical function, the trabecular region supports bone marrow activity, promotes vascularization, and facilitates biochemical communication with adjacent tissues, including the CCL and marrow spaces [30,32].

Structurally, this compartment is characterized by high porosity, marked anisotropy, and substantial metabolic activity. Unlike the more static SBP, the trabecular bone exhibits greater biological activity and responsiveness to mechanical and systemic cues, which may influence its microarchitecture and biochemical signaling pathways [30].

## 3. Development Approaches of Constitutive Models

The development of constitutive models can follow two main approaches. The first is based on theoretical modeling, where a hypothesis is derived from fundamental theories and subsequently validated experimentally to ensure its accuracy and predictive capability [33]. The second is a combined approach, where experimental studies are used to characterize material behavior under mechanical loading, and these empirical observations are translated into mathematical formulations. These models are then refined through computational simulations and validated against experimental data or existing theoretical frameworks [34].

Regardless of the selected approach, the rigorous calibration of the model is crucial. The main material properties, including stiffness, mechanical strength, and microstructural characteristics, must be accurately determined for reliable predictions [35,36].

In this work, we chose to focus on constitutive models, as they align with the theoretical modeling approach, permitting the development of hypothesis-oriented models. Constitutive models are commonly categorized into three main types: (a) strength models, (b) equations of state, and (c) failure models.

### 3.1. Constitutive Models

Constitutive models are mathematical frameworks that describe how materials respond to various mechanical or thermal loading conditions, establishing precise relationships between stress and strain [35]. These models are founded upon fundamental principles such as conservation laws and kinematic relations, translating experimentally observed physical behavior into mathematical equations.

#### 3.1.1. Strength Models

Strength models describe how materials react to mechanical forces, accounting for both elastic and plastic behavior [35]. In the elastic regime, deformation is reversible and follows a linear relationship, as defined by Hooke’s law. However, many materials exhibit elastoplastic behavior, where permanent deformation occurs beyond a certain yield limit. Different yield criteria are applied depending on the material type and loading conditions. The von Mises criterion, for example, is widely used for ductile metals, while models like Mohr–Coulomb and Drucker–Prager are more appropriate for soils and concrete due to their pressure-dependent behavior [33,35]. In cases where the strain rate and temperature significantly influence material response, models such as Johnson–Cook provide a more accurate representation by incorporating these effects [35].

#### 3.1.2. Equations of State (EOS)

EOS describe how a material responds to compression and expansion, playing a critical role in scenarios involving high-pressure conditions such as hypervelocity impacts and explosions. Different EOS formulations exist depending on the nature of the material being studied. For solids subjected to shock waves, the Mie–Grüneisen equation is widely used to relate pressure, volume, and internal energy. In contrast, for highly compressible materials and explosive detonation products, the Jones–Wilkins–Lee equation effectively models the expansion of detonation gases and energy release [35].

#### 3.1.3. Failure Models

Failure models aim to predict the onset and progression of material degradation under load. Failure can be defined using various criteria, such as reaching a critical plastic strain, exceeding a negative hydrostatic stress limit, or surpassing a maximum principal stress threshold. Ductile materials typically fail after accumulating significant plastic strain, whereas brittle materials, such as ceramics and hardened metals, are more prone to failure under excessive tensile stress [33,35]. Additionally, fracture mechanics-based models, such as Griffith’s theory, describe crack growth and propagation in solids. Numerical approaches, such as element erosion in finite element simulations, are also employed to model localized failure [35].

**Table 1:** Quantitative mechanical parameters of osteochondral layers extracted from experimental studies compiled in Berni et al. (2024) [6]. No experimental values were available for healthy SB, CCL, tidemark, or cement line.

Layer	Condition	Elastic or Young’s Modulus (MPa for AC//GPa for SB)	Shear Modulus (MPa)	Poisson’s Ratio	Strain	Equilibrium or Aggregate Modulus (MPa)	Instantaneous Elastic Modulus (MPa)	Initial Fibril Network Modulus (MPa)	Nonfibrillar Matrix Modulus (MPa)	Equilibrium or Aggregate Modulus (MPa)	Initial Permeability (m4/N s)	Viscosity Coefficient (MPas)
AC	Healthy	0.1–0.9 (shear test, compression, linear elastic model, knee) [37] 1.1–3.3 (quasistatic); 0.5–4.98 (0.1 MPa); 40–120 (impact) (compression, linear elastic model, hip) [38] 0.419 ± 0.143 (compression, linear elastic model, knee) [39]	0.01–5.00 (shear test, compression, linear elastic model, knee) [37]	0.00–0.05 (indentation, biphasic, knee) [40]	0.01–0.40 (compressive test); 0.010.50 (shear test) (linear elastic model, knee) [37]	0.48–1.58 (indentation, biphasic model, knee) [40]	0.1–0.4 (indentation, linear elastic isotropic model, knee) [41]			0.90 ± 0.43 (compression, linear elastic model, knee) [42] 0.1–30.0 (tensile, low strain rate); 0.1–70 (tensile, high strain rate)—tensile test, linear elastic, knee, aging [43] 0.9 ± 0.4 (indentation, linear elastic model, knee) [44] 0.48–1.58 (indentation, biphasic, knee) [40]	(1.7–5.4) × 10–15 (indentation, biphasic model, knee) [40]	218.7 ± 150.6 (indentation, viscoelastic model, hip) [45]
	OA	1.0–17.0 (indentation, biphasic model, knee, OARSI grade 0) [46] 1.5–8.0 (indentation, biphasic model, knee, OARSI grade 1) [46] 0.5–9.5 (indentation, biphasic model, knee, OARSI grade 2) [46] 1.0–7.5 (indentation, biphasic model, knee, OARSI grade 3) [46] 1.0–4.5 (indentation, biphasic model, knee, OARSI grade 4) [46] 1.0–2.0 (indentation, biphasic model, knee, OARSI grade 5) [46] 0.69 ± 0.40 (compression, linear elastic model, knee) [47]	0.90 ± 0.10 (indentation, viscoelastic model, knee, ICRS grade 0) [48] 0.57 ± 0.07 (indentation, viscoelastic model, knee, ICRS grade 1) [48] 0.27 ± 0.07 (indentation, viscoelastic model, knee, ICRS grade 2) [48] 0.11 ± 0.05 (indentation, viscoelastic model, knee, ICRS grade 3) [48] 0.16 ± 0.06 (indentation, viscoelastic model, knee, ICRS grade 4) [48]		0.0–0.12 (compression, anisotropic elastic model, knee) [49]	1.2 ± 0.3 (indentation, linear elastic model, knee, early OA) [50] 0.2 ± 0.3 (indentation, linear elastic model, knee, advanced OA) [50]	2.0 ± 1.0 (indentation; compression, linear elastic isotropic; fibril-reinforced poro-viscoelastic, knee, ICRS grade > 0) [51] 4.5 ± 1.0 (indentation; compression, linear elastic isotropic; fibril-reinforced poro-viscoelastic, knee, area surrounding abnormal cartilage) [51] 7.0 ± 1.0 (indentation; compression, linear elastic isotropic; fibril-reinforced poro-viscoelastic, knee, ICRS grade 0) [51] 6.44 ± 4.85 (indentation, linear elastic isotropic; fibril-reinforced poro-viscoelastic model, knee, OARS 0–1) [52] 0.42 ± 1.34 (indentation, linear elastic isotropic; fibril-reinforced poro-viscoelastic model, knee, OARS 2–3) [52] 0.00 ± 0.76 (indentation, linear elastic isotropic; fibril-reinforced poro-viscoelastic model, knee, OARS 4) [52]	0.59 ± 0.48 (indentation, fibril-reinforced poro-viscoelastic model, hip) [53] 0.1–38 (indentation; compression, fibril-reinforced poro-viscoelastic model, knee) [54] 8.5 ± 3.0 (indentation; compression, LEI; FRPVE, knee, ICRS grade > 0) [51] 13.0 ± 2.0 (indentation; compression, linear elastic isotropic; fibril-reinforced poro-viscoelastic, knee, area surrounding abnormal cartilage) [51] 18.5 ± 2.0 (indentation; compression, linear elastic isotropic; fibril-reinforced poro-viscoelastic, knee, ICRS grade 0) [51] 0.41 ± 0.37 (indentation, linear elastic isotropic; fibril-reinforced poro-viscoelastic model, knee, OARS 0–1) [52] 0.07 ± 0.17 (indentation, linear elastic isotropic; fibril-reinforced poro-viscoelastic model, knee, OARS 2–3) [52] 0.002 ± 0.07 (indentation, linear elastic isotropic; fibril-reinforced poro-viscoelastic model, knee, OARS 4) [52]	0.23 ± 0.22 (indentation, fibril-reinforced poro-viscoelastic, hip) [53] 0.1–2.2 (indentation; compression, fibril-reinforced poro-viscoelastic model, knee) [54] 1.2 ± 0.1 (indentation; compression, linear elastic isotropic; fibril-reinforced poro-viscoelastic, knee, ICRS grade > 0) [51] 1.3 ± 0.2 (indentation; compression, linear elastic isotropic; fibril-reinforced poro-viscoelastic, knee, area surrounding abnormal cartilage) [51] 1.1 ± 0.2 (indentation; compression, linear elastic isotropic; fibril-reinforced poro-viscoelastic, knee, ICRS grade 0) [51] 0.35 ± 0.28 (indentation, linear elastic isotropic; fibril-reinforced poro-viscoelastic model, knee, OARS 0–1) [52] 0.10 ± 0.05 (indentation, linear elastic isotropic; fibril-reinforced poro-viscoelastic model, knee, OARS 2–3) [52] 0.05 ± 0.04 (indentation, linear elastic isotropic; fibril-reinforced poro-viscoelastic model, knee, OARS 4) [52]	1.2 ± 0.3 (indentation, linear elastic model, knee, early OA) [50] 0.2 ± 0.3 (indentation, linear elastic model, knee, advanced OA) [50] 0.1–2.2 (indentation; compression, fibril-reinforced poro-viscoelastic model, knee) [54] 0.4–2.4 (indentation, biphasic model, knee, OARSI grade 0) [46] 0.3–1.5 (indentation, biphasic model, knee, OARSI grade 1) [46] 0.2–1.3 (indentation, biphasic model, knee, OARSI grade 2) [46] 0.3–1.4 (indentation, biphasic model, knee, OARSI grade 3) [46] 0.3–1.2 (indentation, biphasic model, knee, OARSI grade 4) [46] 0.2–1.0 (indentation=, biphasic model, knee, OARSI grade 5) [46] 1.19 ± 0.56 (indentation, linear elastic isotropic; fibril-reinforced poro-viscoelastic model, knee, OARS 0–1) [52] 0.42 ± 0.25 (indentation, linear elastic isotropic; fibril-reinforced poro-viscoelastic model, knee, OARS 2–3) [52] 0.21 ± 0.15 (indentation, linear elastic isotropic; fibril-reinforced poro-viscoelastic model, knee, OARS 4) [52]	(3.66 ± 2.86) × 10–15 (indentation, fibril-reinforced poro-viscoelastic model, hip) [45]	36.0 ± 41.4 (indentation, viscoelastic model, hip) [45]
SB	Healthy											
	OA	16.2–24.0 (indentation, elastoplastic model, hip) [55] 15.7–21.1 (indentation, elastoplastic model, hip, severe reported damage) [55] 12.56 ± 0.50 (indentation, linear elastic model, knee, ICRS grade 0) [48] 13.68 ± 0.60 (indentation, linear elastic model, knee, ICRS grade 1) [48] 14.05 ± 0.70 (indentation, linear elastic model, knee, ICRS grade 2) [48] 13.60 ± 1.00 (indentation, linear elastic model, knee, ICRS grade 3) [48] 17.20 ± 2.00 (indentation, linear elastic model, knee, ICRS grade 14) [48]										

## 4. Constitutive Models for Cartilage and Bone

While constitutive models can broadly encompass strength, state, and failure formulations [35], the biomechanical modeling of biological tissue relies predominantly on strength-based models. These focus on describing the stress–strain behavior of materials under mechanical loading, including elastic, plastic, viscoelastic, and poroelastic responses, which are essential for simulating the time- and structure-dependent behavior of living tissues [10,11,33].

**Figure 2:**
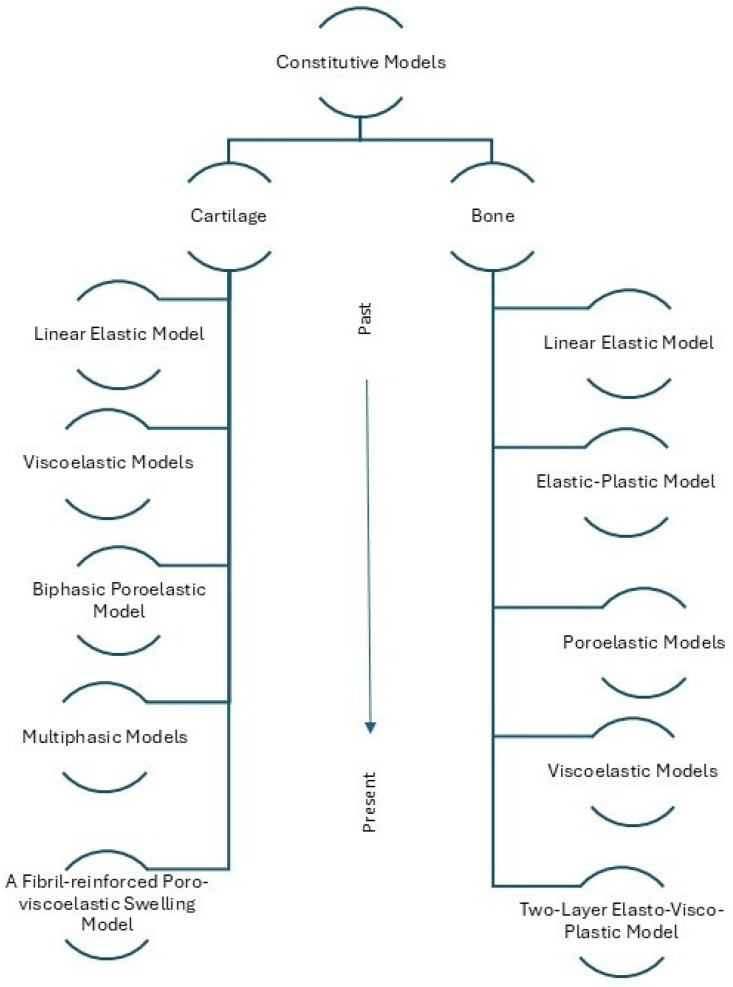
Evolution of constitutive models used in cartilage and bone biomechanics.

Given the inherent complexity and anisotropy of tissues, traditional strength models have been progressively adapted to reflect tissue-specific properties such as fluid–solid interactions, structural heterogeneity, and electrochemical coupling [8,9,34]. The schematic presented on Figure 2 illustrates this conceptual evolution, tracing how foundational mechanical models have been modified and expanded to account for the distinct behaviors of cartilage and bone. From early linear elastic models [36,56] to biphasic [9], fibril-reinforced poro-viscoelastic frameworks [28], and the two-layer elasto-visco-plastic model [57], these developments reflect a growing understanding of tissue biomechanics.

This transition lays the foundation for the integrative OC modeling approaches discussed in the following section. Such approaches aim to simulate the OC unit as a cohesive and mechanically interactive system, thereby enhancing the physiological fidelity and clinical relevance of computational simulations [6,57].

### 4.1. Evolution of Cartilage Models

The modeling of AC biomechanics has evolved significantly in recent decades, driven by advances in both experimental techniques and computational capacities. Early approaches characterized cartilage primarily through simplistic linear elastic models, relying on basic assumptions of homogeneous, isotropic, and elastic behavior [36]. Although computationally efficient, these models failed to capture the complex physiological responses of cartilage under realistic loading conditions.

To address these limitations, Hayes et al. [58] introduced a viscoelastic model, describing cartilage as a time-dependent material capable of exhibiting both immediate elastic response and delayed deformation under load. By incorporating shear and bulk creep behavior, this approach provided a more accurate representation of cartilage mechanics, particularly in capturing the progressive deformation observed under sustained loading.

Mow et al. [9] introduced the biphasic model, a significant advancement in the biomechanical representation of cartilage. This model conceptualized cartilage as a two-phase material, consisting of a porous, elastic solid matrix saturated with interstitial fluid. By incorporating fluid flow and solid deformation interactions, it provided a robust framework for explaining key time-dependent behaviors such as stress relaxation and creep, aligning closely with experimental observations.

Building upon this foundation, Cohen et al. [59] developed the biphasic transversely isotropic model to address the inherent anisotropy of cartilage, primarily dictated by the organization of its collagen network. By introducing transverse isotropy in the solid phase, this model more accurately captured the depth-dependent mechanical properties of cartilage, particularly the directional stiffness variations induced by collagen fibril alignment. This refinement improved the predictive capabilities of biphasic modeling, offering a more physiologically representative description of cartilage mechanics.

Further advancements in cartilage modeling were introduced through multiphasic models, which explicitly incorporated ionic interactions and osmotic swelling phenomena [8,34]. These models accounted for the charged nature of proteoglycans and their role in maintaining cartilage hydration, enabling a more comprehensive representation of biochemical–mechanical coupling. By integrating electrochemical effects into biphasic theory, these models significantly improved the ability to simulate both healthy and degenerative cartilage conditions.

Building upon this foundation, fibril-reinforced poro-viscoelastic models were later developed to further enhance predictive accuracy [28]. These models integrated viscoelastic behaviors of the cartilage matrix, depth-dependent anisotropy, and tension–compression nonlinearity arising from the collagen fibril network. This class of models provided a more physiologically accurate representation of cartilage mechanics under a wide range of dynamic loading scenarios, making them particularly relevant for studying joint function and disease progression.

Overall, the chronological evolution of cartilage modeling reflects an increasing recognition of biological complexity, moving progressively from simple elastic descriptions to sophisticated, physiologically representative multiphasic frameworks. Such advancements have considerably improved our ability to predict cartilage behavior under physiological and pathological conditions, supporting the development of targeted therapeutic interventions. A summary of the key advantages, disadvantages, and evaluated parameters of these models is provided in Table 2.

### 4.2. Evolution of Bone Models

Similarly to cartilage, recent advances in computational technology and experimental techniques have also prompted tremendous advances in the field of bone biomechanics modeling. Early approaches described bone as a homogeneous, isotropic, linear elastic material governed by Hooke’s law [56]. While computationally straightforward, these models oversimplified the true complexity of bone’s structural behavior and mechanical properties.

Advancements in imaging technology, notably micro-computed tomography (micro-CT), enabled the detailed visualization of trabecular bone architecture, prompting the adoption of anisotropic elastic models. These models considered bone as an orthotropic or transversely isotropic material, capturing directional stiffness dependent on trabecular orientation [56]. These models proved to be more suitable for predicting the mechanical behavior of trabecular bone under physiological loads, which is especially relevant in clinical contexts such as osteoporosis and in the assessment of bone structural integrity.

In parallel, elastoplastic models emerged to address limitations of purely elastic models by capturing bone’s yield behavior under higher stress conditions and allowing for better representation of physiological and pathological loading scenarios [11]. These models allowed researchers to simulate permanent deformation and initial damage, providing critical insights into bone’s structural integrity under excessive or repetitive loads.

Further refinements came with the introduction of viscoelastic and poroelastic models. Kafka et al. [10] implemented viscoelastic modeling to capture time-dependent deformation behaviors, including creep and stress relaxation, critical for dynamic loading conditions. Lim et al. [60] went a step further and employed poroelastic models to describe interactions between solid bone matrices and fluid-filled pores, essential for understanding physiological bone fluid dynamics and nutrient transport.

More recently, advanced multiphysics models, combining elastic, plastic, viscous, and structural damage responses, have emerged [57]. These sophisticated models can fully represent the complex mechanical behavior of bone tissue, especially under realistic loading conditions or in pathological situations such as fractures or degenerative diseases. Despite the high predictive accuracy, these models require high computational resources and detailed microstructural characterization, sometimes limiting their immediate practical applicability, but constituting a powerful tool for advanced biomechanical research and for the development of new therapeutic or prevention strategies. A summary of the key advantages, disadvantages, and evaluated parameters of these models is provided in Table 3.

## 5. Integrative Modeling Approaches for Osteochondral Tissues

As described previously, each layer of the OC tissue exhibits unique biochemical composition and mechanical behavior, collectively ensuring joint integrity under physiological loading. While modeling efforts have advanced significantly for both AC and bone [9,11,28,57], less attention has been devoted to the intermediate layers (the CCL, the tidemark, and the cortical SBP) which may be critical in understanding the mechanical coupling and stress transfer across the OC unit.

A recent review [6] emphasized the need for multiscale, integrative models that treat the OC unit as a unified, interactive system rather than as discrete materials. However, implementing such integration often relies on experimental datasets that approximate the composite mechanical response of the OC unit under specific loading conditions. Given the dynamic nature of joint function, these data are typically acquired from animal models or in vitro constructs, and may not fully represent the complex, load-dependent behavior of human tissues. An alternative, and potentially complementary, approach involves leveraging our increasingly detailed understanding of each OC layer to construct modular, layer-specific constitutive models, which could then be systematically integrated into a unified OC model. This strategy aligns well with the natural zonal organization of the OC unit and could enable more physiologically relevant simulations.

Models for the superficial, middle, and deep zones of the AC are well-established, incorporating poroelastic, viscoelastic, and anisotropic properties that reflect their complex load bearing and fluid-regulating functions [28,58,59]. Similarly, the SB has been extensively modeled using linear elastic, elastoplastic, viscoelastic, and poroelastic frameworks [10,11,56,60], with increasing incorporation of microstructural and anisotropic features enabled by high-resolution imaging techniques such as micro-CT [30]. However, it is important to understand the behavior of the middle layers.

### 5.1. Tidemark Modeling Approaches

The tidemark remains underrepresented in computational models despite its structural complexity and functional relevance. Precisely modeling mechanical continuity at this interface poses considerable challenges, given its uneven mineralization levels and variable fibril organization [13].

Experimental studies have demonstrated that tidemark morphology and thickness are sensitive to mechanical stimuli. In particular, load-bearing regions exhibit a highly undulating and thicker tidemark, while non-load-bearing areas present a smoother and thinner interface [62,63].

Given its biomechanical, biochemical, and structural roles, the tidemark could be modeled as a graded viscoelastic interface, capturing both its transitionary mechanical properties and spatial heterogeneity. Interface elements with evolving stiffness, anisotropy, and permeability may provide an appropriate computational framework for representing this unique and dynamic boundary within the osteochondral unit.

Given the biomechanical complexity of the tidemark, we propose a multiscale modeling strategy that allows for different levels of detail and computational complexity, ranging from simplified representations to more refined, structurally informed models.

At the simplest level, the viscoelastic model provides an accessible starting point by capturing the time-dependent behaviors of the tidemark, such as stress relaxation and creep [58]. While it does not represent anisotropy or fluid–solid interactions, its low computational cost makes it suitable for initial multilayer simulations.

A more advanced option is the biphasic transversely isotropic model, as proposed by Cohen et al. [59], which effectively captures the fibrous structure of the tidemark by including anisotropy and fluid–solid interactions. This model reflects the natural orientation of collagen fibers and permeability gradients within this region, offering a good balance between biomechanical accuracy and computational efficiency.

For high-fidelity simulations, the fibril-reinforced poro-viscoelastic swelling model by Wilson et al. [28] offers the most comprehensive approach. This model explicitly incorporates fibril reinforcement, viscoelasticity, anisotropy, and osmotic swelling effects. However, its high complexity and computational demands may pose integration challenges, especially in multilayer simulations.

Overall, the choice of model depends on the specific objectives of the study, allowing flexibility between physiological realism and computational manageability. A comparative summary of the key features of these models is provided in Table 4.

### 5.2. Calcified Cartilage Layer Modeling Approaches

To model the CCL’s mechanical behavior effectively, we suggest a progressive selection of models, depending on the desired balance between complexity and computational feasibility.

As a starting point, the viscoelastic model provides a simplified framework that accounts for time-dependent responses such as stress relaxation. Although it does not capture anisotropy or fluid–solid interactions, it is computationally efficient and well-suited for initial or integrative simulations [58].

The biphasic transversely isotropic model by Cohen et al. [59] offers a more detailed alternative, incorporating anisotropic mechanical behavior and fluid–solid interactions. This makes it especially appropriate for multilayer models aiming to replicate the directional stiffness and permeability of the CCL while maintaining manageable computational costs.

For the most comprehensive and physiologically accurate simulations, the fibril-reinforced poro-viscoelastic swelling model by Wilson et al. [28] explicitly models collagen fibril reinforcement, viscoelasticity, and osmotic swelling phenomena. Despite its high computational demands, this model is ideal for in-depth analyses of the CCL in isolation or in high-resolution contexts.

This range of options allows researchers to tailor their model selection according to specific research goals, ensuring adaptability across different simulation scenarios. The lower boundary of the CCL is defined by the cement line, which serves primarily as a structural anchor. Unlike the tidemark, which is metabolically active and contributes to load modulation and mineralization, the cement line is metabolically inactive. However, it contributes to mechanical stability by anchoring the CCL to the underlying bone, supporting cohesion at the OC interface [12]. A comparative summary of the key features of these models is provided in Table 5.

### 5.3. Subchondral Bone Modeling Approaches

The structural complexity of the SB, encompassing both the SBP and STB, necessitates a tailored modeling approach that captures its heterogeneous mechanical behavior. We propose a progressive modeling strategy that balances physiological accuracy with computational feasibility.

As an initial approach, the linear elastic model offers a practical and computationally efficient option for both the SBP and the STB [56]. The SBP, characterized by its dense, low-porosity structure and isotropic mechanical behavior, can be reliably described using this model, which adequately captures its role as a stiff mechanical bridge between the overlying cartilage and the trabecular network beneath [3]. For the STB, a linear elastic formulation can likewise be adopted, particularly as a first step towards future coupling with the other layers of the osteochondral unit [56]. This choice promotes coherence within the subchondral region and supports the development of integrated multilayer models. However, while this approach offers computational efficiency, it provides only a simplified representation of the STB’s biomechanical complexity. Given that the STB exhibits a highly porous, anisotropic architecture, which plays a critical role in load distribution and shock absorption, more advanced models are required to more accurately capture its mechanical behavior [3,30,32]. Its performance is strongly influenced by microstructural parameters such as bone volume fraction (BV/TV), trabecular thickness, alignment, and interconnectivity [30]. Considering its time-dependent behavior and fluid–solid interactions, the STB is best captured through poroelastic or viscoelastic models [10,60]. These models accommodate the complex biomechanical phenomena of this region, including creep, stress relaxation, and internal fluid flow within the trabecular network. Furthermore, they allow for the integration of anisotropy and microstructural heterogeneity. Comparison between linear elastic, viscoelastic and poroelastic models for SB biomechanical characterization are in Table 6.

### 5.4. Constitutive Model Convergence Across Osteochondral Layers

Defining suitable constitutive models for each layer of the OC unit is essential for developing realistic and integrated simulations of joint biomechanics. As previously discussed, each tissue layer exhibits unique structural, biochemical, and mechanical characteristics. Consequently, a one-size-fits-all modeling approach is inadequate. Instead, a modular, zone-specific strategy should be adopted—one that reflects the intrinsic complexity of each layer while maintaining the flexibility required for future multilayer integration and computational implementation.

Despite their utility, all constitutive models involve trade-offs. Simplified formulations, such as linear elastic or classical viscoelastic models, are attractive for their computational efficiency but often fall short in capturing critical aspects of native tissue behavior, including anisotropy, strain-dependent permeability, and nonlinear responses under multiaxial loading. Conversely, more advanced frameworks—such as fibril-reinforced poro-viscoelastic or swelling-based models—offer improved physiological fidelity but demand substantial experimental input, including the precise characterization of fiber orientation, porosity, and fixed charge density. In most cases, model parameters are derived from in vitro mechanical testing or fitted to experimental stress–strain curves, which may not fully reflect the tissue’s in vivo environment or heterogeneity. Therefore, model selection should be guided by a balance between biological realism, computational demands, and the quality and availability of experimental data.

For the AC, although advanced models such as the fibril-reinforced poro-viscoelastic swelling model proposed by Wilson et al. [28] provide an exceptionally detailed representation of the tissue (accounting for anisotropy, viscoelasticity, porosity, and collagen network mechanics), these models come with substantial computational costs. To facilitate integration within multilayer frameworks, a more pragmatic approach involves starting with the viscoelastic model, as described by Haydes et al. [58]. This model captures the essential time-dependent responses of cartilage, such as creep and stress relaxation, while maintaining computational efficiency. Using the viscoelastic formulation as a foundation enables the robust early-stage integration of the cartilage layers, particularly when exploring interlayer mechanical interactions or conducting sensitivity analyses. As simulation objectives evolve, more sophisticated models like the biphasic transversely isotropic model [59] can be progressively introduced to enhance physiological fidelity.

Given the need for robust integration between layers, for the tidemark and CCL, the viscoelastic model emerges as the recommended starting point for initial coupling studies. This model effectively captures time-dependent behaviors such as creep and stress relaxation, providing an accessible and computationally efficient option for early-stage simulations. Despite its simplicity, the viscoelastic model supports valuable insights into interlayer mechanical interactions, making it ideal for proof-of-concept and exploratory analyses. For more advanced investigations that require higher physiological fidelity, more sophisticated formulations such as the biphasic transversely isotropic model [3] or the fibril-reinforced poro-viscoelastic swelling model can be progressively incorporated. These models enhance the representation of anisotropy, fluid–solid interactions, and fibril reinforcement, allowing for a deeper exploration of tissue-level mechanics and pathological scenarios. However, it is essential to balance the added complexity with the available computational resources and the specific aims of the study.

Given its high stiffness, low porosity, and largely elastic behavior under physiological loads [3], a linear elastic model is sufficient to represent the SBP mechanical response [56]. This model’s simplicity aligns with the structural uniformity of the SBP and minimizes the risk of numerical instability during integration into multilayer frameworks.

For the STB, despite its metabolically active and porous architecture, a linear elastic formulation is also proposed as the initial modeling strategy. While more advanced formulations, such as viscoelastic or poroelastic models [10,60], offer improved physiological accuracy by capturing time-dependent behaviors and anisotropy, the linear elastic model ensures computational efficiency and coherence with the baseline integration approach.

Therefore, we advocate for a flexible and progressive modeling strategy: starting with simplified approaches like linear elastic and viscoelastic formulations to establish robust multilayer integration and subsequently advancing to more complex constitutive laws. This adaptable methodology empowers researchers to tailor their simulations according to the demands of each study, ensuring a balance between computational efficiency and physiological realism.

Table 7 summarizes the characteristics of each OC layer and the suggested computational model for its initial representation. This table provides a practical starting point for the development of integrated OC simulations, establishing a coherent baseline for future model enhancement and progressive refinement.

## 6. Future Directions

**Figure 3:**
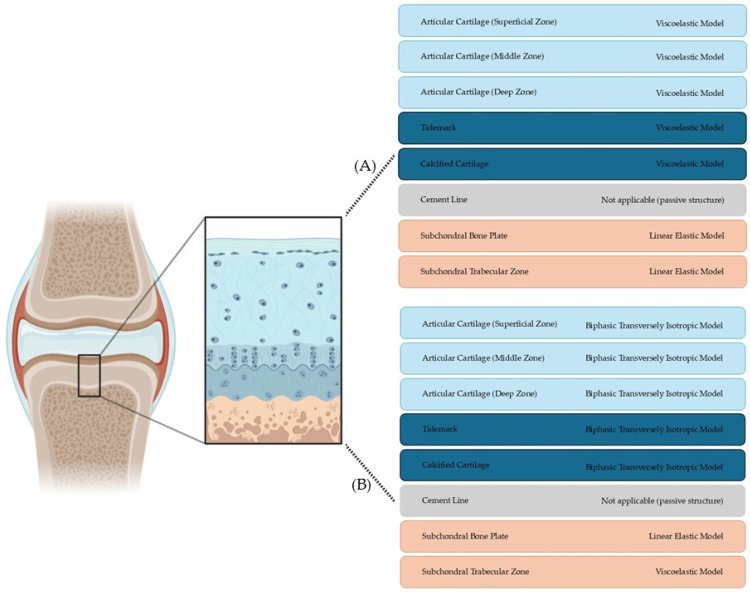
Suggested constitutive models for each layer of the OC unit under a progressive integration strategy. (**A**) illustrates an initial, simplified approach using computationally efficient models to enable early-stage coupling studies. (**B**) depicts an intermediate level modeling approach, incorporating more advanced constitutive laws to improve physiological fidelity.

Although significant progress has been made in characterizing and modeling individual layers of the OC unit, the next essential step lies in integrating these layer-specific models into a unified, computationally coherent framework. Such integration is necessary to simulate the full biomechanical behavior of the OC tissue under both physiological and pathological conditions, as suggested in Figure 3A. This initial framework, based on simplified and computationally efficient models, provides a robust starting point for early coupling studies and proof-of-concept simulations. However, this task presents substantial challenges due to the heterogeneity of material properties, interface complexities, and differing temporal responses across layers.

To achieve higher fidelity in mimicking the complex mechanical behavior of OC tissues, future efforts should progressively evolve toward incorporating more advanced, intermediate-level models that better capture anisotropy, fluid–solid interactions, and time-dependent responses of each tissue layer. This approach, illustrated in Figure 3B, represents an important compromise between computational manageability and physiological accuracy, enabling more realistic simulations of mechanical interactions and degeneration mechanisms across the OC interface.

Future efforts should prioritize the development of multiphysics coupling strategies that allow distinct constitutive models to interact dynamically across well-defined interfaces. This includes implementing finite element (FE) architectures capable of accommodating different responses, as well as managing discontinuities at transitional zones such as the tidemark and the CCL.

Moreover, experimental validation protocols will be critical to ensure the reliability of these integrative models, especially when applied to degeneration processes or scaffold–tissue interactions. Advanced MRI techniques have been instrumental in assessing cartilage repair, offering noninvasive insights into the repair site and surrounding joint tissues [31]. Genetic chondrodysplasias, such as those caused by COL2A1 mutations, offer valuable models for studying the functional roles of specific cartilage zones. For instance, the c.611G>C mutation leads to structural alterations in type II collagen, predominantly affecting the middle and deep zones of the AC. This zonal disruption compromises the cartilage’s mechanical integrity and is associated with early-onset OA, highlighting the critical interplay between genetic factors and zonal cartilage function [65].

The construction of such comprehensive models will not only deepen our understanding of mechanical crosstalk within the OC unit but will also provide robust in silico platforms for preclinical exploration. These tools could significantly enhance the design of biomimetic scaffolds, the evaluation of material–tissue integration, and the prediction of disease progression in controlled laboratory settings.

By tackling these challenges, future integrative models will deepen our understanding of osteochondral tissues and speed up clinical translation, paving the way for earlier diagnosis and personalized treatments for OA.

## 7. Conclusions

This review underscores the complexity of OC tissues and the critical importance of adopting integrative modeling strategies that reflect their layered structure and distinct biomechanical roles. Rather than treating the OC unit as a homogeneous material or simulating its components in isolation, a more physiologically accurate approach involves defining layer-specific constitutive models that are both experimentally grounded and computationally compatible.

After reviewing the existing models and discussing their suitability for each OC layer, we propose the most appropriate starting points for capturing their mechanical behavior. For the cartilaginous regions, including the tidemark and the CCL, the viscoelastic model is suggested as a pragmatic choice for initial integration studies. Supported by foundational experimental work such as that of Hayes et al. [58], this model effectively captures time-dependent phenomena like creep and stress relaxation, while maintaining manageable computational demands. A linear elastic model [56] remains sufficient for both the SBP and the STB, reflecting the dense, stiff nature of the SBP and offering a pragmatic simplification for the trabecular bone that ensures coherence and computational efficiency in early-stage multilayer integration.

This modular modeling architecture allows for improved biomechanical continuity across tissue interfaces, while remaining flexible enough to incorporate future refinements based on emerging imaging, biochemical, and mechanical data. Importantly, this strategy facilitates the construction of multilayer finite element models capable of capturing physiological loading conditions, pathological alterations, and the mechanobiological processes involved in tissue degeneration and repair.

Ultimately, the development of such integrative models provides a robust foundation for advancing research on OC tissue. By enabling high-fidelity in silico simulations and supporting the design of biomimetic materials for experimental testing, these models open new avenues for investigating the mechanical behavior of healthy and diseased OC structures. This approach may significantly enhance our ability to explore degeneration mechanisms, validate engineered scaffolds, and simulate interventions under controlled laboratory conditions, contributing to the evolution of OC research in both basic science and preclinical domains. In the future, such in silico models may also play a key role in reducing the use of animal models, promoting more ethical and sustainable research practices.

## Figures and Tables

**Table 2:** Detailed comparison of primary constitutive models used to represent the mechanical behavior of cartilage.

Model	Key Principle	Advantages	Disadvantages	Parameters	Author(s)
Isotropic Linear Elastic Model	Linear elastic response to loading	Simple mathematical formulation and easy to implement; Requires minimal experimental input for parameter estimation; Suitable for analyzing indentation tests with different probe geometries; Provides a first approximation of cartilage mechanical properties.	Assumes isotropic and purely elastic behavior, ignoring viscoelasticity; Does not account for fluid flow or swelling effects; Limited accuracy for complex loading conditions; Less representative of actual cartilage microstructure.	Young’s modulus Poisson’s ratio Stress and deformation in cartilage	Hayes et al. [36]
Viscoelastic (Generalized Kelvin–Voigt Model)	Time-dependent deformation (viscoelastic solid behavior)	Captures time-dependent behavior (creep and stress relaxation); Simple formulation with experimentally validated parameters; Applicable to physiological load ranges.	Does not explicitly include fluid flow or poroelastic effects; Limited accuracy for high-frequency loading; Assumes homogeneity and isotropy.	Young’s modulus Poisson’s ratio Shear modulus	Hayes et al. [58]
Biphasic Model	Solid–fluid interaction (poroelastic behavior with fluid flow through porous solid)	Simple and widely used in biomechanical studies; Effectively captures creep and stress relaxation behavior; Experimentally validated.	Does not account for ionic effects or swelling behavior; Assumes constant permeability, which may not be accurate in all cases; Does not explicitly include the role of collagen fibers	Permeability Aggregate modulus Frictional drag coefficient Relaxation time Solid matrix stiffness	Mow et al. [9]
Biphasic Transversely Isotropic Model	Anisotropic poroelastic behavior (fiber-reinforced solid–fluid interaction)	Provides a more accurate representation of the mechanical behavior of cartilage by incorporating transverse isotropy, which is essential for understanding the anisotropic nature of the tissue; Improves the correlation between theoretical predictions and experimental results in unconfined compression tests.	Increases computational complexity due to the additional parameters required to characterize anisotropy; Demands detailed experimental data to accurately determine the anisotropic material properties.	Young’s modulus Poisson’s ratio Permeability Shear modulus	Cohen et al. [59]
Triphasic Model	Solid–fluid–ionic coupling (electrochemical and poroelastic effects)	Includes electrochemical effects such as fixed charge density and osmotic pressure; Describes interactions between solid matrix, interstitial fluid, and ionic phase; Accounts for swelling and ion transport effects.	More complex and computationally demanding; Requires additional experimental parameters for validation; Assumes a single salt solution for ion exchange, which may oversimplify real conditions.	Osmotic pressure Fixed charge density Ion concentration gradients Permeability Solid matrix stress–strain properties	Lai et al. [8]
Quadriphasic Model	Multiphase coupling (electro-chemical–mechanical interaction)	Incorporates fluid flow, ion transport, and electrical effects for a more realistic tissue response; Captures swelling behavior and incompressibility of biological tissues; Suitable for modeling cartilage and soft tissues under physiological conditions; Provides insights into electrochemical interactions affecting tissue mechanics.	Highly complex and computationally demanding; Requires extensive experimental data for parameter calibration; Difficult to validate experimentally due to multiple interacting phases; Less intuitive and harder to implement than simpler elastic or biphasic models.	Solid matrix stress and deformation Fluid pressure and flow Ion concentration distribution Electrical potential gradients	Huyghe et al. [34]
Fibril-Reinforced Poro-viscoelastic Swelling Model	Fiber-reinforced, poro-viscoelastic behavior with osmotic swelling	Includes collagen fiber reinforcement and viscoelastic behavior; Captures swelling effects and fibril anisotropy; Can describe multiple experimental tests (confined/unconfined compression, indentation, and swelling).	Highly complex and computationally intensive; Requires detailed structural information about collagen fiber orientation; More difficult to parameterize experimentally.	Collagen network stiffness Viscoelastic properties of fibrils Poroelasticity and swelling parameters Hydraulic permeability Chemical expansion stress	Wilson et al. [28]

**Table 3:** Detailed comparison of primary constitutive models used to represent the mechanical behavior of bone.

Model	Key Principle	Advantages	Disadvantages	Parameters	Author(s)
Linear Elastic Model	Linear elastic response to loading	Simple formulation based on Hooke’s law, easy to implement in numerical simulations; Suitable for small strain conditions typically observed in physiological loading; Requires only a few mechanical parameters; Allows straightforward comparison across experimental and computational studies; Efficient for simulating early-stage mechanical responses of trabecular bone.	Does not capture post-yield behavior or progressive damage of bone tissue; Assumes homogeneity and isotropy, which may not reflect real trabecular structure; Inaccurate under large deformations or in cases of bone failure; Ignores time-dependent effects such as creep or stress relaxation; May oversimplify complex biomechanical conditions.	Young’s modulus Poisson’s ratio Stress Strain	Cowin et al. [56]
Transversely Isotropic Model	Directional elasticity (anisotropic response based on trabecular orientation)	Captures the anisotropic mechanical behavior of trabecular bone more accurately than isotropic models; Provides a better representation of trabecular bone under physiological loading conditions; Enhances the precision of finite element models in biomechanics applications.	Requires detailed experimental data to define material properties along different axes; Increased computational complexity compared to isotropic models; Variability in trabecular microstructure can lead to challenges in generalizing model parameters.	Young’s modulus (longitudinal and transverse) Shear modulus Poisson’s ratio Elastic coefficients	Brown et al. [61]
Elastoplastic Model	Elastic–plastic transition (includes yield behavior under load)	Captures both elastic behavior and the onset of permanent deformation; Reflects the yield behavior of trabecular bone under uniaxial loading; Allows estimation of yield strain independently of trabecular orientation; Supports simplified modeling using an isotropic yield criterion; Facilitates correlation between yield properties and bone density measures.	Depends on a defined offset criterion, which may vary across studies; Focuses on compression only, limiting broader biomechanical application; Does not model post-yield behavior such as plastic flow or damage accumulation; May not represent accurately the behavior of human or pathological bone; Assumes isotropy, which may not hold true for all trabecular structures.	Young’s modulus Yield stress Yield strain Bone tissue density Solid volume fraction Degree of trabecular orientation	Turner et al. [11]
Viscoelastic Model	Time-dependent deformation (viscoelastic response with internal stress relaxation)	Captures both elastic and time-dependent (viscous) behavior of trabecular bone; Models the composite nature of trabecular bone, accounting for internal architecture; Reflects anisotropic mechanical response under different loading directions; Differentiates between dynamic and static behavior; Provides a solid theoretical foundation for analyzing creep and stress relaxation.	Involves complex equations and numerous parameters, increasing model complexity; Assumes simplified conditions in practical applications; Limited by the availability of experimental data to calibrate all variables accurately; May be less suitable for real-time simulations or clinical applications due to computational intensity.	Young’s modulus Viscosity coefficient Structural volume fractions Structural anisotropy parameters Dynamic and static moduli Internal stress and strain tensors Relaxation behavior under step-loading	Kafka et al. [10]
Poroelastic Model	Poroelasticity (coupled solid deformation and fluid flow through porous structure)	Accounts for the interaction between the bone matrix and internal fluids; Can be applied to simulate realistic physiological conditions of trabecular bone; Provides greater accuracy in predicting the mechanical behavior of bone under uniaxial loading.	Requires parameters that are difficult to measure experimentally; Complex modeling that is computationally intensive; May need adjustments for different bone types and specific biomechanical conditions.	Young’s modulus Shear modulus Poisson’s ratio Skempton’s coefficient Permeability coefficient Pore pressure	Lim et al. [60]
Two-Layer Elasto-Visco-Plastic Model	Multimechanism coupling (elasticity, viscosity, and plastic deformation)	Accounts for both viscoelastic and plastic behavior of bone tissue, providing a more realistic model; Allows for the accurate identification of the rheological parameters of bone; Applicable for modeling bone deformations under various loading conditions.	Requires complex mechanical testing for parameter calibration; More sophisticated mathematical modeling, making it computationally demanding; May need adjustments for different bone tissue types and specific biomechanical conditions.	Young’s modulus Viscosity coefficient Poisson’s ratio Plasticity parameters	Reisinger et al. [57]

**Table 4:** Comparison between viscoelastic, biphasic transversely isotropic, and fibril-reinforced poro-viscoelastic swelling models for tidemark biomechanical characterization.

Feature	Viscoelastic Model	Biphasic Transversely Isotropic Model	Fibril-Reinforced Poro-Viscoelastic Swelling Model
Anisotropy (fiber orientation)	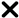	✔	✔
Fluid–solid interactions (poroelasticity)	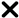	✔	✔
Viscoelasticity	✔	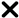	✔
Fibril reinforcement	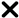	Implicit	Explicit
Osmotic swelling	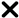	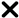	✔
Computational complexity	Low	Moderate	High
Ease of integration	High	High	Lower

**Table 5:** Comparison between viscoelastic, biphasic transversely isotropic, and fibril-reinforced poro-viscoelastic swelling models for CCL biomechanical characterization.

Feature	Viscoelastic Model	Biphasic Transversely Isotropic Model	Fibril-Reinforced Poro-Viscoelastic Swelling Model
Anisotropy (fiber orientation)	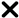	✔	✔
Fluid–solid interactions (poroelasticity)	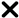	✔	✔
Viscoelasticity	✔	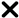	✔
Fibril reinforcement	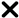	Implicit	Explicit
Osmotic swelling	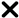	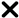	✔
Computational complexity	Low	Moderate	High
Ease of integration	High	High	Lower

**Table 6:** Comparison between linear elastic, viscoelastic and poroelastic models for SB (SBP and STB) biomechanical characterization.

Feature	Linear Elastic Model (SBP and STB)	Viscoelastic Model (STB)	Poroelastic Model (STB)
Anisotropy (fiber orientation)	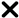	Implicit	✔
Fluid–solid interactions (poroelasticity)	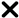	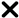	✔
Viscoelasticity	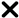	✔	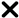
Microstructural representation	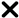	Implicit	✔
Time-dependent behavior	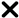	✔	✔
Computational complexity	Low	Moderate	High
Ease of integration	High	High	Lower

**Table 7:** Summary characterization of OC tissue layers.

Layer	Description	Material Type	Suggested Model
AC (Superficial Zone)	Contains type II collagen fibers aligned parallel to the joint surface; provides resistance to shear stress [14,15,16].	Porous, viscoelastic soft tissue	Viscoelastic model [58]
AC (Middle Zone)	Disorganized collagen fibers; high proteoglycan content; dissipates and distributes initial compressive loads [14,15,16].	Porous, viscoelastic soft tissue	Viscoelastic model [58]
AC (Deep Zone)	Contains type II collagen fibers oriented perpendicular to the articular surface; provides strong anchorage to the calcified cartilage and resists high compressive loads during weight-bearing activities [14,15,16].	Porous, viscoelastic soft tissue	Viscoelastic model [58]
Tidemark	Fibrillar band separating unmineralized from CCL; regulates force transmission; structurally adapted to loading [15,19,20,23,26,61,62,63].	Graded viscoelastic interface	Viscoelastic model [58]
CCL	Mineralized zone between the tidemark and cement line; transitional mechanical layer; contains type I and II collagen and hypertrophic chondrocytes [12,15,24,25,26,27,64].	Stiff, mineralized fibrocartilage	Viscoelastic model [58]
Cement Line	Defines the lower boundary of CCL; acts as a structural anchor without metabolic activity [12].	Structural boundary (passive anatomical feature)	Not applicable (passive structure) [12]
SBP	Dense cortical bone layer beneath CCL; distributes load to underlying trabecular bone [3].	Dense cortical bone	Linear elastic model [56]
STB	Porous trabecular bone network responsive to mechanical stimuli; supports load, vascularization, and marrow communication [3,30,32].	Porous trabecular bone	Linear elastic model [35]

## Data Availability

The data that support the findings of this study are available from the corresponding author on request.

## Abbreviations

The following abbreviations are used in this manuscript:

AC	Articular Cartilage
BV/TV	Bone-Volume-to-Total-Volume Ratio
CCL	Calcified Cartilage Layer
EOS	Equations of State
OA	Osteoarthritis
OC	Osteochondral
SB	Subchondral Bone
SBP	Subchondral Bone Plate
